# Bioenergetic Pathways in the Sperm of an Under-Ice Spawning Fish, Burbot (*Lota lota*): The Role of Mitochondrial Respiration in a Varying Thermal Environment

**DOI:** 10.3390/biology10080739

**Published:** 2021-08-01

**Authors:** Deepali Rahi, Borys Dzyuba, Tomas Policar, Oleksandr Malinovskyi, Marek Rodina, Viktoriya Dzyuba

**Affiliations:** South Bohemian Research Center of Aquaculture and Biodiversity of Hydrocenoses, Faculty of Fisheries and Protection of Waters, Research Institute of Fish Culture and Hydrobiology, University of South Bohemia in Ceske Budejovice, Zátiší 728/II, 389 25 Vodňany, Czech Republic; bdzyuba@frov.jcu.cz (B.D.); policar@frov.jcu.cz (T.P.); omalinovskyi@frov.jcu.cz (O.M.); rodina@frov.jcu.cz (M.R.); vdzyuba@frov.jcu.cz (V.D.)

**Keywords:** oxidative phosphorylation, glycolysis, fatty acid oxidation, sperm motility, spawning temperature, maximum critical temperature, cold-water fish

## Abstract

**Simple Summary:**

The burbot (*Lota lota*) is the only endangered or threatened freshwater gadoid that usually spawns in icy waters (<6 °C), and whereas the sperm bioenergetics of many fish species have been studied in the context of adaptation to warmer environments, the sperm of cold-water fish are the least explored. Therefore, this study was undertaken to determine both the roles of the most important energy-supplying pathway(s) in the burbot before and after sperm become motile at the spawning temperature, and the mitochondrial adaptation at the maximum temperature that is tolerable. The results reveal that burbot sperm have a naturally low oxygen consumption rate (respiration) and a limited capacity for enhancement under exposure to an uncoupler. Oxidative phosphorylation is more realized near the critical thermal tolerance limit. However, similar to the sperm of most other freshwater species, this pathway, which occurs during motility, is insufficient to fulfill the large energy demands of the motile sperm. Therefore, be it at the spawning temperature or at a higher temperature, the majority of the energy required for motility is derived from pre-stored ATP reserves produced during a quiescent state.

**Abstract:**

Regarding the sperm of cold-water fish, the contributions of different bioenergetic pathways, including mitochondrial respiration, to energy production at the spawning temperature and its adaptation at the maximum critical temperature (CTmax) are unclear. The roles of glycolysis, fatty acid oxidation, oxidative phosphorylation (OXPHOS) at 4 °C, and OXPHOS at 15 °C for energy production in burbot (*Lota lota*) spermatozoa were studied by motility and the oxygen consumption rate (OCR) (with and without pathway inhibitors and the OXPHOS uncoupler). At both temperatures, the effects of the inhibitors and the uncoupler on the motility duration, curvilinear velocity, and track linearity were insignificant; in addition, the OCRs in activation and non-activation media differed insignificantly and were not enhanced after uncoupler treatment. After inhibitor treatment in both media, OXPHOS was insignificantly different at the 2, 30, and 60 s time points at 4 °C but was reduced significantly at the 30 and 60 s time points after treatment with sodium azide at 15 °C. In conclusion, for burbot sperm at both the spawning temperature and the CTmax, the energy synthesized via OXPHOS during motility was insufficient. Therefore, the majority of the energy required to sustain motility was derived from pre-accumulated energy produced and stored during the quiescent state of the spermatozoa.

## 1. Introduction

The burbot (*Lota lota*) is a holarctic, cold-water stenotherm that undertakes long migrations during the spawning season (mid-winter) and reproduces externally [1], meaning the temperature affects its life cycle and fertilization. The optimum feeding temperature, optimum spawning temperature, and maximum critical temperature (CTmax) are 12 °C, 1–4 °C (often under ice), and 12–14 °C, respectively (the fish avoids temperatures > 14 °C) [2]. Therefore, the physiological functions of this cold-water fish are realized in a narrow temperature range: 1–15 ± 1 °C.

In an externally fertilizing fish, sperm are released into the water and remain motile for only a moment. Motility, a prerequisite for successful fertilization, is regulated by enzymes, including those involved in energy-supplying pathways. Generally, the relation between enzymatic activity and temperature is a classic bell curve, but unlike the “classic rule,” widely accepted by fish spermologists, a different observation was recorded for burbot sperm. For example, after the temperature changed from 4 to 12 and 20 °C, the key energy-supplying enzymes (ATPase, adenylate kinase, pyruvate kinase, and malate dehydrogenase) showed reduced activity [3]. Since the burbot is a species that spawns in icy conditions, it offers a rare opportunity to study the energetic pathways of fish sperm at extremely low temperatures and their adaptations to enhanced temperature.

It is commonly accepted that most of the energy required to maintain adequate ATP for motility in fish is generated through mitochondrial respiration; however, mitochondrial respiration’s contribution to energy production might vary with the temperature. The general rule implies that as the temperature rises, the prevalence of sperm motility and sperm velocity increase because of an enhanced respiration rate due to higher enzymatic activity. Contrary to the general rule though, these enhancements are sometimes caused even by lower temperatures [4,5,6]. This phenomenon, well known in some ectotherms, is referred to as “thermal compensation” [7] and has been studied mostly in somatic tissues. The present study, however, focuses on the gamete (sperm).

In the sperm of fish that fertilize externally, it is commonly recognized that the energy for motility activation and prolongation is produced in the form of ATP during a quiescent but bioenergetically active state, stored, and then expended after activation. Various energy-supplying pathways (glycolysis, phospholipid catabolism, triglyceride metabolism, the Krebs cycle, and oxidative phosphorylation (OXPHOS)) are recruited to produce energy for motility. Nevertheless, the dominant contribution of any of these at a particular motile or immotile state is debatable, species-specific, and follows no general trend [8]; moreover, different spawning temperatures make the generalization even more complicated.

To the best of our knowledge, no study has been conducted on the sperm mitochondrial respiration role (before and during sperm motility) for energy production in fish that spawn in extremely cold water and have special emphasis on mitochondrial activity that adapts to temperature increases. Whether such adaptations are species-specific or can be generalized or extrapolated from somatic tissue studies cannot be distinguished with any certainty.

Furthermore, when the coupling of substrate oxidation and ATP synthesis is incomplete, a mitochondrial anomaly called “proton leak” occurs, and the respiration rate rises but does not generate energy (ATP). In various externally fertilizing fish, such as the cold-water trout (*Oncorhynchus mykiss*) and the warm-water carp (*Cyprinus carpio*) and turbot (*Psetta maxima*), studies have evaluated the enhancements in respiration during active and inactive states [8]. It has been revealed that the spermatozoa of some fish enhance oxidative metabolism after motility activation or after being exposed to an uncoupling agent [8,9,10]. Despite this documented knowledge, no prediction has yet been made for the enhanced respiration in a particular fish species based on its thermal habitat or taxon.

Although the sperm mitochondrial function has been explained for several fish species from varying thermal regimes, the only extreme cold water data, thus far, concern the trout, which spawns at 10 °C [11]. Therefore, this study was designed to reveal the following for burbot sperm: (a) the relative contribution of specific energy-supplying pathways (glycolysis, fatty acid oxidation, and OXPHOS) in activated and non-activated states at a spawning temperature of 4 °C; (b) changes in the role of the most important energy-supplying pathway when the environmental temperature approaches the critical limit (CTmax 15 °C); and (c) any significant enhancements in oxidative metabolism (in the presence of an uncoupler or after motility activation) at the spawning temperature or CTmax. The results were then compared with the known trends of various fish with distinct thermal preferences (cold water vs. warm water).

## 2. Materials and Methods

### 2.1. Broodstock, Spermiation, and Sperm Collection

Experiments were conducted in compliance with the principles of the Ethics Committee for the Protection of Animals in Research of the University of South Bohemia in Ceske Budejovice. Three-year-old adult male broodstock (spawning season: January–February; average body weight ~250 g) were cultured in a circular holding tank (40–60 individuals per 500 L tank) at the Faculty of Fisheries and Protection of Waters, University of South Bohemia, Vodnany, Czech Republic. The tank’s temperature was kept below 5 °C, and the photoperiod mimicked the preferred natural spawning habitat: 9 h light, 15 h dark. Sperm were stripped by abdominal massage, collected into a 3 mL syringe, kept on ice, and then transferred to a laboratory. As a quality control, only sperm samples having motility >80% were accepted.

### 2.2. Basal Solutions for Activation and Non-Activation Media

The buffered sperm motility activation medium (AM) used for the burbot consisted of 50 mM NaCl, 10 mM Tris-HCl, and 1 mM CaCl_2_. The buffered sperm non-activation medium (NAM) comprised 100 mM NaCl, 40 mM KCl, 10 mM Tris, and 1 mM CaCl_2_. The pH was adjusted to 8.5. Osmolality was 110 mOsmol/kg for AM, and 290 mOsmol/kg for NAM.

### 2.3. Reagents Used in the Experiment—Inhibitors and an Uncoupler

The inhibitors 2-deoxy-d-glucose (DOG), sodium fluoride (NaF), and sodium azide (NaN_3_) were used to inhibit the key enzyme of glycolysis (glucose-6-phosphatase [12]), fatty acid oxidation (fatty acid oxidase, enolase [13]), and OXPHOS (cytochrome oxidase [14]), respectively. Additionally, to assess the enhanced respiration rate in active and non-active burbot sperm, the oxygen consumption rate (OCR) was studied by exposure of sperm to an OXPHOS uncoupling agent, carbonyl cyanide m-chlorophenyl hydrazine (CCCP) [15]. The concentration of every inhibitor (DOG, NaF, and NaN_3_) was 1 mM, and that of the uncoupler (CCCP) was 1 µM. The concentrations of the reagents were standardized after conducting a preliminary experiment. The pH and osmolality of both AM- and NAM-containing reagents were confirmed to be unchanged prior to the experiment.

### 2.4. Motility Assessment

A dominant energy contribution of any of the energetic pathways during the motile state was determined by analyzing motility parameters in AM without (control) and with each inhibitor (DOG, NaF, and NaN_3_) and the uncoupler (CCCP). For this, a 40 µL drop of AM (with or without reagents) containing 0.25% pluronic acid was placed onto a microscope slide, and sperm were added using the tip of an insulin syringe needle and thoroughly mixed to a dilution of ~1:10,000 [16] to obtain the appropriate concentration for analysis. All motility records were taken at 4 ± 1 °C. Additionally, to evaluate the OXPHOS activity at a higher temperature, motility with and without OXPHOS inhibitor and uncouplers (NaN_3_ and CCCP) was studied at 15 ± 1 °C.

A thermoblock (HLC BO50/15, Landsberger, Berlin, Germany) was used to adjust the temperatures of the solutions. The microscope was equipped with a cooling stage (Olympus IX83, Southend-on-Sea, United Kingdom). The adjusted temperature was monitored via a copper-constantan thermocouple (Omega, L-044T, Taipei, Taiwan) using a data logger thermometer (Omega, HH127, Taipei, Taiwan). Video records were created microscopically under a 10× lens and a negative phase-contrast condenser with an Imaging Development Systems (IDS) digital camera equipped with uEye Cockpit software set at 25 frames·s^−1^. Video records were saved in avi format, and analysis was conducted with an Integrated System for Semen Analysis (ISAS software; PROISER, C/ Catedrático Agustín Escardino, Paterna, Spain) at 10, 20, 30, 40, 50, and 60 s, and at 10, 20, 30, and 40 s post-activation at 4 and 15 °C, respectively. Sperm curvilinear velocity (VCL), linearity of track (LIN), and motility duration were selected as the studied parameters for both temperature groups. Sperm having a velocity less than 10 µm·s^−1^ were considered to be non-motile. Motility duration was recorded from each video from the beginning of motility until 95% of the spermatozoon stopped moving. The stepwise methodology used to record motility and further analysis are provided in Supplementary Figure S1.

### 2.5. Sperm Concentration and Measurement of Oxygen Consumption Rate

Sperm concentration was evaluated by using a Burker cell hemocytometer (Meopta, Kabelikova 1, Prerov, Czech Republic) and an Olympus BX 50 phase-contrast microscope (200× magnification; Olympus, Shinjuku, Tokyo, Japan) [17].

The OCR of burbot sperm was measured with a Clark-type polarographic oxygen probe (YSI 5300A Biological Oxygen Monitor; Brannum Lane Yellow Springs, OH, USA) immersed in a chamber with a water jacket (regulating the temperature to 4 or 15 °C) kept on a magnetic stirrer (frequency of rotation 800 rpm). For assessing the influences of inhibitors of energy-supplying pathways (DOG, NaF, and NaN_3_) on the sperm OCR, the chamber was filled (1000 µL) with media (AM or NAM) with or without (control) inhibitors and then closed with an oxygen probe. A 50 µL sperm sample was injected through the insertion hole a few seconds after obtaining a stable signal. For the assessment of the influence of the CCCP uncoupler on the OCR, a sperm sample was injected in a closed chamber containing media (AM or NAM without an uncoupler), and a reagent was then injected 120 s after the injection of sperm. Additionally, to study the role of OXPHOS at a higher temperature, the OCR was measured in the presence and absence of an inhibitor and uncoupler (NaN_3_ and CCCP) of OXPHOS in AM and NAM at 15 °C.

With a polarographic system, oxygen saturation data (%) at each 2 s interval were obtained in real time. Taking into account the cell concentration for each male and the solubility of oxygen at different temperatures, the final OCR was calculated from the oxygen content (%) for each group (males, media combinations, temperature, and time). For this, OCR was calculated every 2 s (from 0 to 72 s) while keeping a 10 s tracking interval (0–10, 2–12, 4–14, etc., up to 62–72 s). Finally, to demonstrate the OCR changes occurring in the presence and absence of inhibitors at 0–70 s, average values from five males and two replicates for each group were calculated. The dots obtained from the averaged values were connected and plotted on a line graph.

Additionally, to analyze the effects of inhibitors on the OCR of burbot sperm, statistical analysis was performed (elaborated in Section 2.6) at the beginning, middle, and end of motility (2, 30, and 60 s, respectively). The effect of the uncoupler at each temperature (4 and 15 °C), in each medium (AM and NAM), was determined by calculating the OCRs at the 3rd min (120–180 s) in the presence and absence of CCCP at 1 µM concentration. All the calculated values are expressed in nmol O_2_ min^−1^ (10^9^ spz)^−1^. The general experimental design and workflow are presented in Supplementary Figure S2.

### 2.6. Statistical Analyses

Statistical analyses for each group (temperature, media combinations (AM/NAM with and without reagents), and time points were conducted in STATISTICA v12 (Statsoft Inc., Hillview Avenue Palo Alto, CA, USA). Each experimental value was obtained by averaging the results of five male fish with two replicates each. The mean values of the males were calculated and used for statistical analyses. Interactions were considered statistically significant at *p* ≤ 0.05.

The data distribution characteristics and homogeneity of dispersion were evaluated by using the Shapiro–Wilk test and Levene’s test, respectively. A parametric test, one-way ANOVA followed by Tukey’s honest significant difference (HSD), was applied for the normally distributed data with similar dispersion values. For the abnormally distributed data or data with an absence of homogeneity (OCR values at 15 °C), a non-parametric test (Mann–Whitney test) was applied to analyze the effect of the inhibitor in the presence of the respective media (AM with AM + NaN_3_ and NAM with NAM + NaN_3_).

## 3. Results

### 3.1. Sperm Motility Parameters

**Figure 1:**
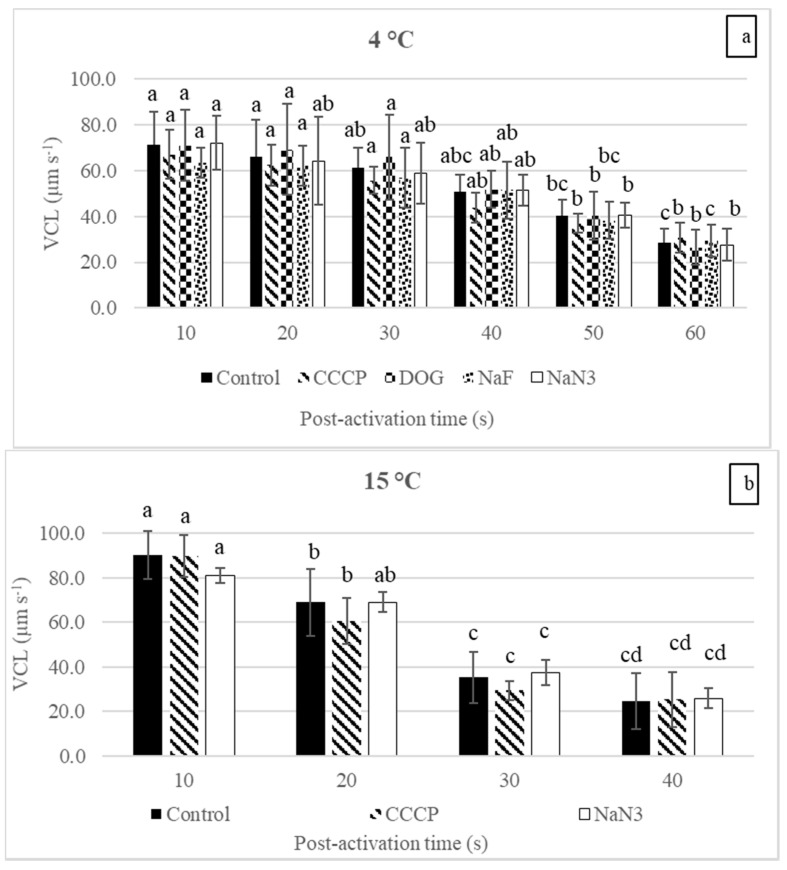
The influences of inhibitors and an uncoupler on the curvilinear velocity (VCL) of burbot (*L. lota*) sperm at different post-activation time points in AM at (**a**) 4 °C; (**b**) 15 °C. Control, no reagent; CCCP, carbonyl cyanide m-chlorophenyl hydrazine; NaF, sodium fluoride; DOG, 2-deoxy-D-glucose; NaN_3_, sodium azide. Inhibitors and the uncoupler were used at 1 mM and 1 μM concentrations, respectively. Values with different letters at different post-activation times are significantly different (*p* < 0.05, Tukey’s HSD test). Data are presented as mean ± S.D.

**Figure 2:**
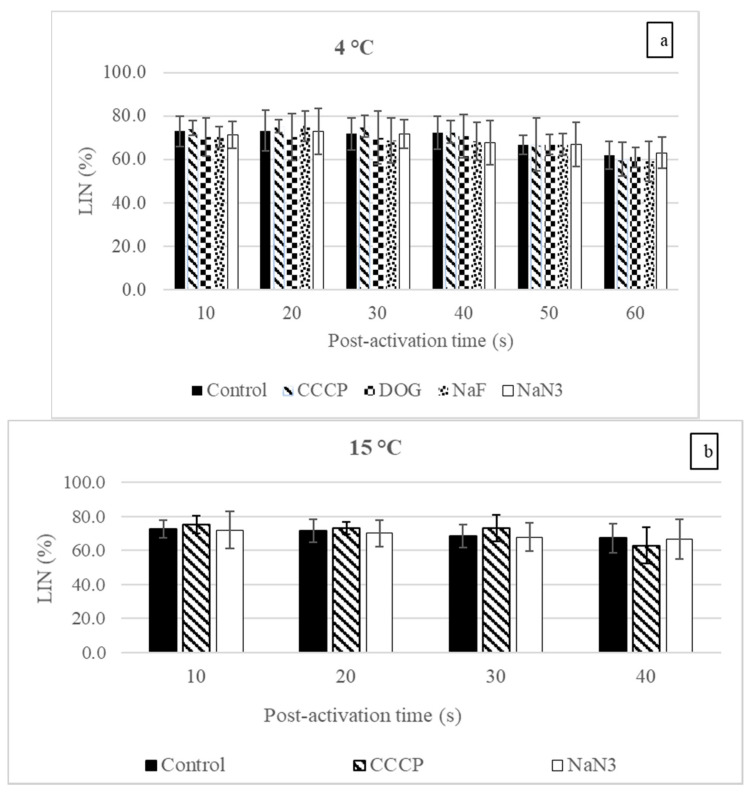
The influences of inhibitors and an uncoupler on the linearity of track (LIN) of burbot (*L. lota*) sperm at different post-activation time points in AM at (**a**) 4 °C; (**b**) 15 °C. Control, no reagent; CCCP, carbonyl cyanide m-chlorophenyl hydrazine; NaF, sodium fluoride; DOG, 2-deoxy-D-glucose; NaN_3_, sodium azide. Inhibitors and the uncoupler were used at 1 mM and 1 μM concentrations, respectively. At 4 and 15 °C, no significant changes were observed either after treatment with any reagents or past the post-activation time (*p* > 0.05, Tukey’s HSD test). Data are presented as mean ± S.D.

At 4 and 15 °C, the reduction in the burbot sperm VCL after treatment with each reagent (inhibitors and uncoupler) at any post-activation time point was insignificant (Figure 1a,b). Similar results were obtained for the LIN (Figure 2a,b).

In the control, the reduction in the VCL post-activation was slow at 4 °C and decreased significantly only at 50 s compared with 10 s (Figure 1a). At 15 °C, the reduction in the VCL over the post-activation time was faster than at 4 °C (the VCL was significantly reduced at 20, 30, and 40 s compared with 10 s (Figure 1b)). No significant changes were observed in the LIN at any time point at 4 and 15 °C (Figure 2a,b). Additionally, after raising the temperature from 4 to 15 °C, the VCL at 10 s post-activation was significantly increased in the control and CCCP groups (1.27 and 1.33 times, respectively) (*p* < 0.05, one-way ANOVA), but no significant enhancement was observed in the NaN_3_ group (*p* > 0.05, one-way ANOVA).

At 4 and 15 °C, the motility duration in the control group was insignificantly changed after treatment with any studied reagent (*p* > 0.05, one-way ANOVA). At 15 °C, the motility duration was significantly reduced compared to 4 °C in all groups: control, NaN_3_, and CCCP—1.40, 1.38, and 1.45 times, respectively (*p* < 0.05, one-way ANOVA) (Table 1).

### 3.2. Oxygen Consumption Rate (OCR)

**Figure 3:**
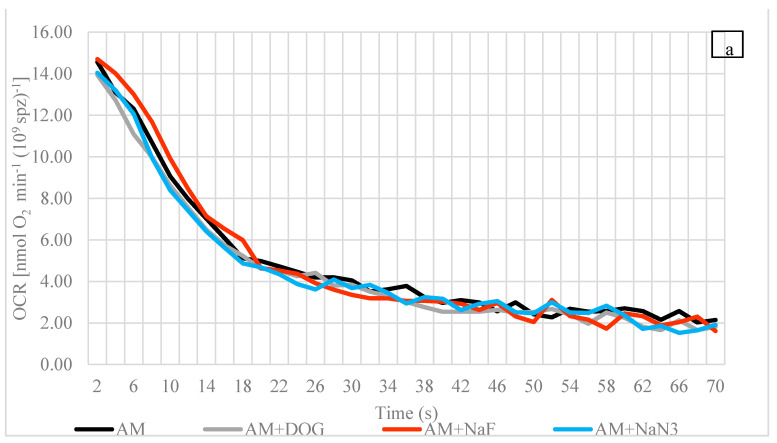

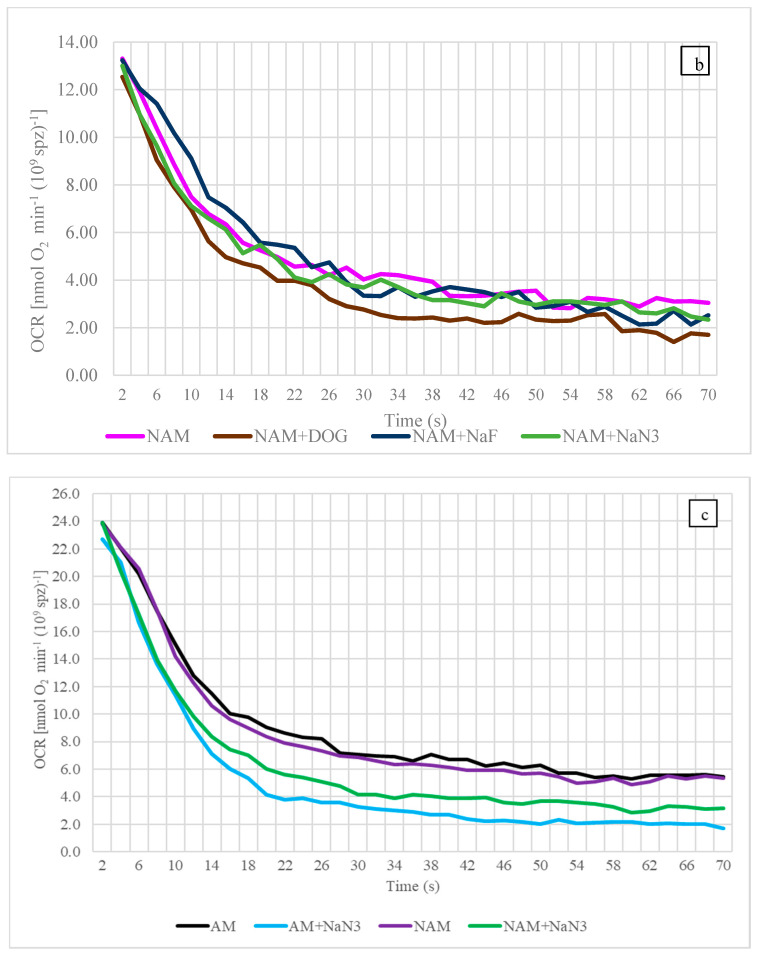
Oxygen consumption rate (OCR, nmol O_2_ min^−1^ (10^9^ spz)^−1^) of burbot (*L. lota*) sperm at (**a**) 4 °C in AM; (**b**) 4 °C in NAM; (**c**) 15 °C in AM and NAM. Line plots were created with and without inhibitors at 0–70 s. Each line was obtained by connecting the dots of average values for combinations of male/medium composition/time. AM, activation medium; NAM, non-activation medium; AM + NaF, activation medium containing sodium fluoride; AM + DOG, activation medium containing 2-deoxy-D-glucose; AM + NaN_3_, activation medium containing sodium azide; NAM + NaF, non-activation medium containing sodium fluoride; NAM + DOG, non-activation medium containing 2-deoxy-D-glucose; NAM + NaN_3_, non-activation medium containing sodium azide. Values of OCR at 2, 30, and 60 s are presented in Table 2.

At 4 °C, as with the situation at 15 °C, the OCRs in AM and NAM at all time points (2, 30, and 60 s) were insignificantly different from each other (Figure 3a–c). In addition, at 4 °C, no significant change in the OCR compared to the control was found after treatment with any inhibitor at 2, 30, or 60 s in either medium (AM or NAM) (Figure 3a,b).

At 15 °C, in each medium, no significant effect of NaN_3_ at 2 s was observed compared to the control (*p* > 0.05, Mann–Whitney U test). At 30 and 60 s, a significant reduction in the OCR after exposure to NaN_3_ was observed in AM and NAM (*p* < 0.05, Mann–Whitney U test) (Figure 3c, Table 2). The OCR significantly increased at 15 °C compared to 4 °C in AM and NAM at 2 (1.71 and 1.90 times), 30 (1.77 and 1.75 times), and 60 s (1.89 and 1.63 times) (*p* < 0.05, one-way ANOVA). No significant changes were observed after exposure to an uncoupling agent (CCCP) in AM or NAM at 4 or 15 °C.

## 4. Discussion

The burbot is the only freshwater gadoid that is endangered or threatened in North America and Eurasia [18,19]. Increased temperatures due to climate change are one reason among many, such as pollution, urbanization, and invasive non-native species, behind the declining population of the burbot. Several studies have been conducted to mitigate the population decline directly or indirectly. Most of them have focused on understanding its life cycle, enhancing reproduction in hatcheries, or understanding and improving sperm physiology [3,16,20,21,22,23,24]. Nevertheless, sperm energy budgeting (production, accumulation, and usage) and its adaptation to raised temperatures were still unexplored before this study commenced. This article is the first report on the determination of the dominant energy-supplying pathway (glycolysis, fatty acid oxidation, or mitochondrial respiration) that contributes to the energy production required for sperm motility at its spawning temperature. Additionally, the modulation in the role of OXPHOS (the most important bioenergetic pathway in most freshwater species) was studied at CTmax (15 °C). Furthermore, the obtained results at the spawning temperature and CTmax were compared to published data on various freshwater species that spawn in different thermal regimes.

Since it is the only freshwater species belonging to the order Gadiformes, the burbot has retained many characteristics of ancestral marine and cold stenothermal fish, such as high fecundity and a pelagic larval stage [21]. Nevertheless, the spermatozoon motility duration (which is known to be generally shorter for freshwater species and longer for marine fish) at 4 °C was typical of other freshwater species such as the Arctic char (*Salvelinus alpinus*) and the pikeperch (*Sander lucioperca*) in temperatures of 2–5 °C [25,26]. The VCL was well within the range of previous values found for the burbot, but lower than the VCL of trout sperm [20,27,28].

### 4.1. Influences of the Inhibitors and Uncoupler on Sperm Motility at Spawning Temperature

The relative contributions of energy-supplying pathways have been debated for decades, not only for fish spermatozoa but also for mammals [29,30]. An additional question often asked among fish spermologists is whether fish sperm generate and use energy during motility or produce energy (ATP) before motility and then use only pre-stored energy. Most studies on fish found that energy generation during sperm motility was the least contributing factor [31,32,33]. Thus, the majority of energy required was derived from a pre-accumulated source produced while in a quiescent state. However, a handful of studies on trout presented contradictory results that carried the debate forward [34].

In sperm bioenergetics, determining the most important energy-supplying pathways by studying the influences of inhibitors and uncouplers on sperm motility or the OCR has been going on for decades. The reagents used in this study (NaN_3_, CCCP, DOG, and NaF) were previously used widely on animals belonging to different taxa [35,36,37,38]. The results reveal that at 4 °C, the sperm VCL and LIN were insignificantly changed after exposure to any inhibitor at any post-activation time (Figure 1a and Figure 2a). Similar results were observed for the motility duration (Table 1). In line with the present study, the insignificant effect of the OXPHOS inhibitor (potassium cyanide, KCN) was observed on the sperm swimming velocity of the common carp [33]. In the zebrafish (*Danio rerio*), OXPHOS inhibitor (KCN) and an uncoupler (2,4 DNP) did not inhibit the sperm motility percentage until 90 s, which was more than its motility duration [32]. In the Danube bleak (*Chalcalburnus chalcoides*), insignificant changes in sperm motility, linearity, nonlinearity, circular swimming motion, and viability were observed after treatment with DOG or NaF [35]. These data indicate that burbot sperm are metabolically depressed and have no major contribution from any studied pathway during motility at the spawning temperature (icy conditions). The results also suggest that the burbot adopts the energy budgeting strategy adopted by most fish because no energy-supplying pathway provides enough energy to maintain motility during the active state. Thus, most of the energy was derived from pre-accumulated energy produced during a quiescent but metabolically active state.

An altogether different approach is adopted by the spermatozoa of another warm-water species, the African catfish (*Clarias gariepinus*), which uses glycolysis, lipid catabolism, and fatty acid oxidation for energy production during motility [36]. The catfish is rare among other teleosts in that these pathways contribute to energy production during sperm motility. While teleost species (cyprinids and salmonids, having been intensively studied) have the metabolic capacity for glycolysis, oxidative phosphorylation, and lipid metabolism [35,39,40,41], the contributions of these pathways for energy supply during motility are the least known [42].

The formation of reactive oxygen species (ROS), mainly during biological processes such as respiration, is a common cellular phenomenon. Metabolic byproducts such as superoxide radicals (O_2_•−), hydrogen peroxide (H_2_O_2_), hydroxyl radicals (•OH), and singlet oxygen (^1^O_2_) are common ROS species that are formed during phosphorylation reactions and oxygen metabolism. They eventually manifest into oxidative stress [43]. At higher temperatures, when the OCR increases substantially in either motile or quiescent sperm, there is a concomitant increase in ROS production [44]. As lipids are perhaps the most prone to oxidation, even at low temperatures, they are the largest contributor of ROS [45]. Fortunately, cellular systems can detoxify themselves [43]. Burbots have a ω6/ω3 fatty acid (FA) ratio, which is more skewed towards ω6 than ω3: 1.6:1 to ~3:1 (re-calculated from [20]). Such a proportionately high level of ω6 FAs is known to slow down lipid oxidation and ROS formation [46,47]. Therefore, burbot sperm may be naturally predisposed to protect themselves from ROS attacks. This study did not characterize ROS formation at the high temperatures where the OCR increases, but future efforts may be directed towards understanding this mechanism.

### 4.2. The Influence of Temperature on Sperm Motility

There is a conventional opinion that the level of enzymatic activity increases with temperature to a certain point. Any further increases in temperature reduce activity. Thus, we have a bell curve. Additionally, among fish spermologists, it is well known that when the temperature rises, the sperm velocity, motility rate, flagellum beat frequency, and ATP consumption rate are enhanced, leading to a shorter motility duration [48]. Nevertheless, some fish species behave in the opposite way [3,49,50]. The burbot spermatozoon VCL at 10 s post-activation and motility duration increased and decreased significantly, respectively, with a rise in the temperature. The results of the motility parameters are aligned with those of previous studies conducted on numerous species, including the burbot, which showed a higher motility percentage when the temperature ranged from 5 to 20 °C [23,51]. The results suggest that the motility parameters (VCL and motility duration) for the burbot follow the general rule.

An increase in the velocity with an increase in the temperature was not observed when NaN_3_ inhibited mitochondrial respiration (Figure 1a,b). Additionally, in the control group, the reduction in the VCL over the post-activation time at 15 °C was faster than at 4 °C. The above-mentioned results clearly indicate predominant ATP production (via OXPHOS) and faster consumption at a higher temperature. These findings are in agreement with a study conducted on carp, a warm-water fish, that showed that the decrease in the ATP content during motility is faster at 20 °C than at 2 °C [33].

### 4.3. Oxygen Consumption Rate

Recently, to measure the OCRs in biological samples, several novel methodologies have been devised, among which are the MitoXpress fluorescent assay [52], electron paramagnetic resonance oximetry [53], the Seahorse metabolic assay [54], the scanning electrochemical microscopy method [55], and the respiratory detection system [56]. Nevertheless, most mitochondrial respiration studies in fish spermatozoa, including this one, were performed using the conventional Clark-type polarographic method [10,31,35,36,57].

To gain a better understanding of the bioenergetic processes during the most important phase of fish spermatozoa, i.e., motility, ATP content, or the OCR of the spermatozoa of several fish, species have been investigated from the first moments of motility (0–60 s) [33,52,58,59]. In this study, at 4 °C, burbot sperm OCRs in AM and NAM at 2 s were 14.6 ± 3.3 and 13.3 ± 2.6 nmol O_2_ min^−1^ (10^9^ spz)^−1^, respectively. Within 30 s, the AM and NAM values dropped dramatically by 3.60 and 3.33 times, respectively, and then remained similar until the final point of study at 60 s (Figure 3a,b; Table 2). The relatively low (compared to 2 s) but unaltered values of the OCR in AM and NAM during motility (30 to 60 s) were comparable to those of another cold-water species, the trout [11,60]. The burbot OCRs we measured, accompanied by the trout values, lie in the lower range of data for teleosts (1 to 280 nmol O_2_ min^−1^ (10^9^ spz)^−1^) [8]. In contrast to these cold-water species, a very high OCR, followed by a sharp fall within 2 min, was observed for the turbot. This trend was observed only in AM. In NAM, a constant OCR was observed from 0 to 12 min [31]. The results of this study suggest that OXPHOS in motile burbot sperm at the spawning temperature (ice conditions) occurs at a very low level and does not account for any major contribution to energy production during motility.

Fish sperm, in general, are metabolically depressed, which is common for cold-water species. The OCR values at all studied times (2, 30, and 60 s) did not vary significantly between AM and NAM (Figure 3a,b; Table 2). This observation is supported by the majority of the data for the trout [11,57,59]. In contrast, the trend of an enhanced OCR after motility activation is common in temperate, warm-water, or marine species such as the carp, African catfish, Siberian sturgeon (*Acipenser baerii*), and turbot [9,10,31,36]. To the best of our knowledge, the burbot in the presented study and the trout mentioned above are the only cold-water species in which spermatozoon metabolic pathways have been studied by means of the OCR. Therefore, based on limited data, the conclusion that the mitochondrial OCR of a cold-water species has no capacity for enhancement after motility begins is not strong enough, and numerous intensive cold-water fish studies are needed.

### 4.4. Influences of Reagents (Inhibitors and Uncoupler) on OCR at Spawning Temperature

At 2, 30, and 60 s, no significant inhibitor influence on the burbot sperm OCR was observed at 4 °C in AM or NAM (Figure 3a,b; Table 2). These results support the findings for motility (VCL, LIN, and duration) (Figure 1a and Figure 2a). In contrast, the negative effects of mitochondrial inhibitors on the sperm OCR or its outcome, ATP, have been observed in numerous marine and freshwater species, including the common carp, the rainbow trout (*Oncorhynchus mykiss*), the turbot, and the gilthead seabream (*Sparus aurata*), suggesting the existence of ATP production via mitochondrial respiration during motility. However, due to the low capacity of OXPHOS during motility, the major energy source is pre-stored ATP [31,33,57,59,61]. Other cold water-spawning species, such as the rainbow trout, also metabolize lipids when in a non-active state. Those lipids are available as intra- and extra-cellular materials which could aid in sperm motility [62,63], but we did not observe lipid oxidation to be a statistically significant energy-supplying pathway in an active or non-active state, even though lipids have the highest calorific value (9 kcal·g^−1^).

Existing data on the lipid composition of burbot sperm indicate that the ratio of polyunsaturated fatty acids (PUFAs)/saturated or mono-unsaturated fatty acids (SFAs + MUFAs) is ~1:1 [20]. Although mitochondrial β-oxidation in fish happens more freely for SFAs and MUFAs, β-oxidation of PUFAs can be quite variable or complicated [64]. One of the reasons that burbot sperm mitochondrial β-oxidation was not significant is that the lipid composition was not completely skewed towards “β-oxidation labile” SFAs + MFAs but was in equilibrium with PUFAs (more complicated for β-oxidation).

In this study, the OCRs in AM and NAM at 4 and 15 °C were not enhanced after uncoupler treatment. The results are similar to the data for the trout at 10 °C [11,57]. While warm-water species such as the carp, sturgeon, and turbot spawn at 15–20 °C, enhanced OCRs after uncoupling in a non-active state have been observed [9,10,31]. The results suggest a homologous pattern for an uncoupling effect in the spermatozoa of cold-water (no enhanced capacity) and warm-water fish (enhanced capacity). However, the lack of background studies on cold-water fish species prevents us drawing the conclusion that cold-water fish have no capacity for enhanced mitochondrial oxidative metabolism.

### 4.5. The Influence of Temperature on OCR

At 15 °C, the increases in the sperm OCR in AM and NAM at each time point (2, 30, and 60 s) were significant. However, as with the results for the VCL, the decrease from 2 to 60 s was much faster at 15 °C (23.9 to 5.3 nmol O_2_ min^−1^ (10^9^ spz)^−1^ in AM, and 23.5 to 4.9 nmol O_2_ min^−1^ (10^9^ spz)^−1^ in NAM) than at 4 °C (14.6 to 2.7 nmol O_2_ min^−1^ (10^9^ spz)^−1^ in AM, and 13.3 to 3.1 nmol O_2_ min^−1^ (10^9^ spz)^−1^ in NAM). In line with the results of this study, more than double the OCR in African catfish sperm was observed in AM and NAM when the temperature rose from 18 to 28 °C [36]. However, the rule of an enhanced OCR (and its outcome, ATP) with raised temperature is not universal. For example, in a non-active state, the ATP content of carp sperm remained unchanged when comparing 2 and 20 °C [33].

Apart from fish spermatozoa, numerous studies have also been conducted on various tissues and organs to explain the thermal response of mitochondrial functioning. In contrast to the above-mentioned thermal response or “general rule,” a greater mitochondrial density, higher OCRs, and higher mitochondrial enzymatic activity have all been observed at low temperatures [4,5,6]. This phenomenon is well known in some ectotherms and is referred to as “thermal compensation” [7]. No such response was seen in this study. Altogether, it seems that the adaptation of mitochondrial functioning to varying thermal conditions is species-specific and cannot be generalized based on the thermal habitat.

Furthermore, at 15 °C, at 30 and 60 s, there were significantly lower OCRs in AM and NAM compared to the control after exposure to NaN_3_, an inhibitor of OXPHOS (Table 2). Thus, this study reveals that, in the burbot, the role of mitochondrial respiration in energy production becomes more pronounced at high temperature, whereas it is barely detectable (low OCR and no NaN_3_ inhibition) at low temperature. However, to more deeply explain the adaptation strategies in the sperm mitochondria of cold-water fish at high temperatures, detailed studies of the changes in mitochondrial morphology and membrane potential, and of modulations in the energy-supplying pathways other than OXPHOS at different temperatures, are the footsteps for the future.

We compared the sperm energetics of the burbot at its CTmax with that of the Siberian sturgeon—using a similar study published by our research group [10]—because the burbot’s CTmax is exactly at the Siberian sturgeon’s spawning temperature. Even at the same temperature (15 °C), the sturgeon’s motility duration (a few minutes) was longer than that of the burbot (<1 min).

The burbot sperm OCR at 15 °C, 60 s post-activation (5.3 ± 0.8 nmol O_2_ min^−1^ (10^9^ spz)^−1^: the last studied time point for the burbot but the first for the sturgeon), was 2.5 times lower than that of the sturgeon (13.2 ± 1.0 nmol O_2_ min^−1^ (10^9^ spz)^−1^) at the same temperature [10]. Similar results were obtained in a non-activation mediums: burbot, 4.9 ± 1.9 nmol O_2_ min^−1^ (10^9^ spz)^−1^; sturgeon, 9.0 ± 0.4 nmol O_2_ min^−1^ (10^9^ spz)^−1^. Additionally, in burbot sperm, as in Siberian sturgeon sperm, OXPHOS was inhibited by NaN_3_ in AM and NAM (Figure 3c) [10]. Although the sperm mitochondrial activity was enhanced and played an important role in the energy production of our cold-water fish at this “high” temperature, the OCR level did not reach the level attained by temperate fish at the same temperature. The results show evidence of mitochondrial adaptation in the sperm of fish acclimatized to a particular thermal regime.

Furthermore, there might be a relationship among the number and size of mitochondria and the bioenergetic requirements of sperm. For example, salmonids have a low OCR range (0.6–3.9 nmol O_2_ min^−1^ (10^9^ spz)^−1^) and one mitochondrion or a pair of mitochondria, whereas sturgeon sperm, which has a higher OCR range (8.5–13 nmol O_2_ min^−1^ (10^9^ spz)^−1^), has a greater number of mitochondria (3–6) [8,10,65,66]. A similar quantitative study of burbot sperm mitochondria will reveal detailed bioenergetic features.

### 4.6. Future Directions

Future studies may also include assessments of the direct molecular turnover of amino acids (e.g., de novo bioconversions of creatine from arginine and arginine from proline [67,68]) or fatty acids (e.g., in PUFAs/SFAs + MUFAs and ω6/ω3 fatty acid ratios [64,69,70]), or of the metabolizable losses (catabolism) of amino and fatty acids when producing energy [71]. Considering the recent arguments surrounding NaF as an ineffective glycolysis inhibitor [72,73] (see discussions in [10,74,75]), additional techniques, such as evaluating the mitochondrial membrane potential, could provide clarity.

To the best of our knowledge, this study is the first to explain the relative contributions of energy-supplying pathways in a fish species that spawns in icy conditions. Determining the role of mitochondrial respiration in the burbot at higher temperatures and comparing the results with those of species with varied thermal regimes helped explain the basics of mitochondrial functioning in fish spermatozoa in different thermal habitats.

## 5. Conclusions

The results on sperm motility and the OCR observed at 4 and 15 °C suggest that burbot spermatozoa at the spawning temperature (ice conditions) are metabolically depressed with a low OCR and no capacity to enhance oxidative metabolism either by motility activation or by uncoupler treatment. The role of OXPHOS became more prevalent at CTmax; however, at both temperatures, the energy generated via OXPHOS during motility was not efficient enough to fulfil the high energy demand. Therefore, irrespective of the temperature, the energy production and usage strategy remained the same: most of the spermatozoon energy was derived from stored ATP that had been synthesized via OXPHOS during a quiescent but bioenergetically active state.

## Figures and Tables

**Table 1:** Effects of inhibitors (at 1 mM) and an uncoupler (at 1 µM concentration) on the motility duration of burbot (*L. lota*) sperm in an activation medium at 4 °C and 15 °C.

Exposures	4 °C (s)	15 °C (s)
Control	65.8 ± 5.1 a	46.7 ± 1.8 b
CCCP	63.0 ± 5.4 a	45.6 ± 2.6 b
NaF	67.1 ± 3.1	-
DOG	68.7 ± 4.2	-
NaN_3_	68.2 ± 5.3 a	47.0 ± 5.9 b

Control, no reagent; CCCP, carbonyl cyanide m-chlorophenyl hydrazine; NaF, sodium fluoride; DOG, 2-deoxy-D-glucose; NaN_3_, sodium azide. Values with different letters within rows are significantly different (*p* < 0.05, Tukey’s HSD test). Data are presented as mean ± S.D.

**Table 2:** Effects of inhibitors (at 1 mM concentration) on oxygen consumption rate (nmol O_2_ min^−1^ (10^9^ spz)^−1^) of burbot *(L. lota*) sperm at 2, 30, and 60 s in AM and NAM.

Exposures	2 s	30 s	60 s
**4 °C**			
AM	14.6 ± 3.3 a	4.0 ± 0.8 a	2.7 ± 1.0 a
AM + NaF	14.7 ± 2.7 a	3.3 ± 1.3 a	2.6 ± 0.9 a
AM + DOG	13.8 ± 2.3 a	3.9 ± 1.9 a	2.3 ± 0.6 a
AM + NaN_3_	14.0 ± 4.1 a	3.7 ± 1.5 a	2.4 ± 1.8 a
**4 °C**			
NAM	13.3 ± 2.6 a	4.0 ± 1.0 a	3.0 ± 0.8 a
NAM + NaF	13.2 ± 4.1 a	3.3 ± 1.5 a	2.5 ± 1.2 a
NAM + DOG	12.5 ± 3.0 a	2.8 ± 0.7 a	1.9 ± 0.4 a
NAM + NaN_3_	13.0 ± 2.9 a	3.7 ± 1.6 a	3.1 ± 1.2 a
**15 °C**			
AM	23.9 ± 5.2 a	7.1 ± 1.4 a	5.3 ± 0.8 a
AM + NaN_3_	22.7 ± 5.1 a	3.2 ± 0.7 b	2.1 ± 0.6 b
**15 °C**			
NAM	23.5 ± 8.8 a	6.5 ± 2.2 a	4.9 ± 1.9 a
NAM + NaN_3_	23.9 ± 8.1 a	4.2 ± 2.0 b	2.9 ± 1.6 b

AM, activation medium; NAM, non-activation medium; AM + NaF, activation medium containing sodium fluoride; AM + DOG, activation medium containing 2-deoxy-D-glucose; AM + NaN_3_, activation medium containing sodium azide; NAM + NaF, non-activation medium containing sodium fluoride; NAM + DOG, non-activation medium containing 2-deoxy-D-glucose; NAM + NaN_3_, non-activation medium containing sodium azide. In columns, no significant changes are observed in AM and NAM after treatment with any reagent at 4 °C (*p* > 0.05, Tukey’s HSD test). In each column, values with different letters represent differences at 15 °C (*p* ≤ 0.05, Mann–Whitney U test). Data are presented as mean ± S.D.

## Data Availability

The data presented in this study are available on request from the corresponding author.

## Supplementary Materials

The following are available online at https://www.mdpi.com/article/10.3390/biology10080739/s1, Figure S1: Method used for motility assessment, Figure S2: General experimental plan.
