# Supplementary figures and images for: Bioenergetic Pathways in the Sperm of an Under-Ice Spawning Fish, Burbot (Lota lota): The Role of Mitochondrial Respiration in a Varying Thermal Environment
Source: Biology (Basel). 2021 Aug 1;10(8):739. doi: 10.3390/biology10080739 (PMC8389567; doi:10.3390/biology10080739)

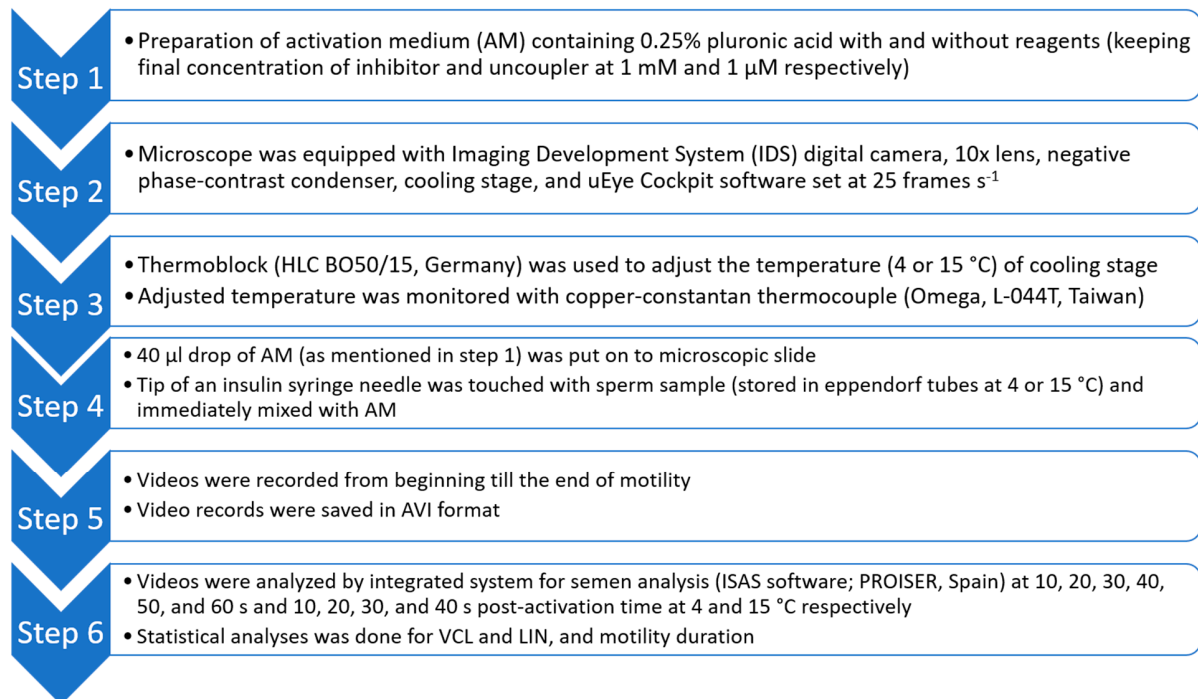

**Figure S1.** Method used for motility assessment

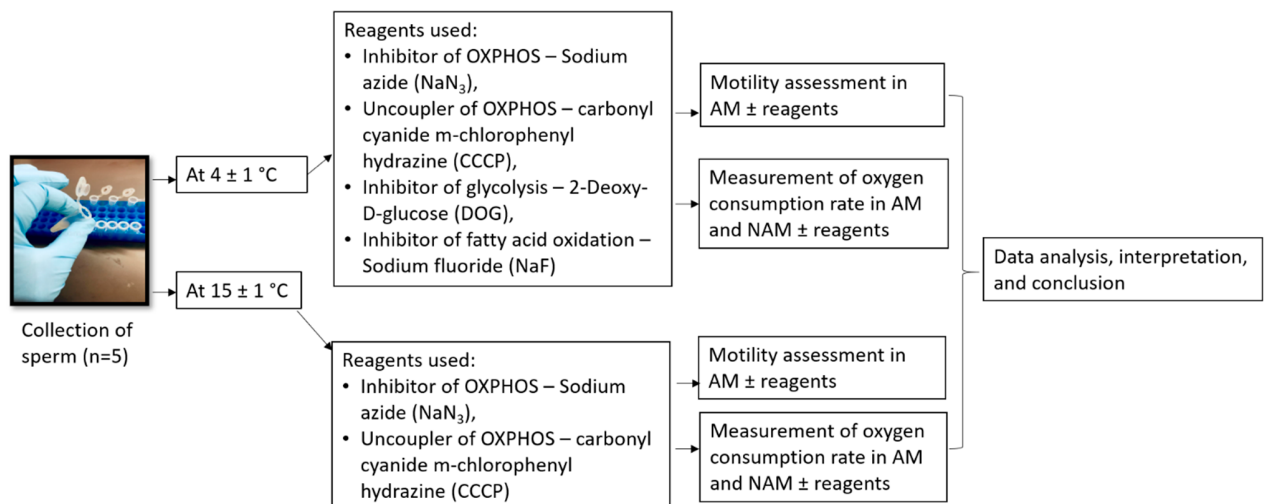

**Figure S2.** General experimental plan

Supplement: Supplementary file 1 [file biology-10-00739-s001.zip › biology-1271183-supplementary.pdf]
